# Supplementary material for: Gamification as a health education strategy of adolescents at school: Protocol for a systematic review and meta-analysis
Source: PLoS One. 2023 Nov 30;18(11):e0294894. doi: 10.1371/journal.pone.0294894 (PMC10688727; doi:10.1371/journal.pone.0294894)
Supplement: S1 File — (DOC) [file pone.0294894.s002.doc]

**Supporting information [Draft of Search Strategy]**

| **DATABASE** | **RESEARCH QUESTION (RQ)** | **EQUATIONS** |
| --- | --- | --- |
| EMBASE | RQ1* | (adolescent OR teenager OR student) AND (gamification OR school OR health education) |
| EMBASE | RQ2** | (adolescent OR teenager OR student) AND (gamification OR school OR health education) AND (diet OR food and nutrition OR dairy products) |
| EMBASE | RQ3*** | (adolescent OR teenager OR student) AND (gamification OR school OR health education) AND (sleep quality OR dyssomnias OR sleep disorders OR sleep hygiene) |
| EMBASE | RQ4**** | (adolescent OR teenager OR student) AND (gamification OR school OR health education) AND (exercise OR sedentary behavior) |
| RQ1* What are the main intervention strategies with gamification techniques for the health education of adolescent students? RQ2** What are the effects of using gamification interventions on sleep quality parameters in adolescent students? RQ3*** What are the effects of using interventions with gamification on the food consumption parameters of adolescent students? RQ4**** What are the effects of using gamification interventions on the physical activity parameters of adolescent students? | | |
