# Supplementary material for: Gamification as a health education strategy of adolescents at school: Protocol for a systematic review and meta-analysis
Source: PLoS One. 2023 Nov 30;18(11):e0294894. doi: 10.1371/journal.pone.0294894 (PMC10688727; doi:10.1371/journal.pone.0294894)
Supplement: Attachment — Submitted filename: Response to Reviewers.docx [file pone.0294894.s004.docx]

**Federal University of Rio Grande do Norte**

**Post-Graduation Program in Public Health**

Av. Senador Salgado Filho, 1787 – Lagoa Nova

ZIP: 59056-000 – Natal, RN, Brazil

July 3^rd^, 2023

Rebuttal Letter for PLOS ONE

Dear Oriel Jerome Delas Alas Vida,

Thank you for considering our manuscript for publication at PLOS ONE. We are certain of the credibility and commitment that PLOS ONE has to quality in science, and we hope that the modifications made in our manuscript fully meet the publication criteria.

We have sure that the suggestions add quality and clarity to our paper. We list at the end of this letter the answers for each one of them.

Once again, thank you for considering our manuscript and we are at your disposal for any further information.

Sincerely,

The authors.

**Authors’ responses to the Editor (Email June 30^th^, 2023)**

1. **Please review your reference list to ensure that it is complete and correct. If you have cited papers that have been retracted, please include the rationale for doing so in the manuscript text or remove these references and replace them with relevant current references. Any changes to the reference list should be mentioned in the rebuttal letter that accompanies your revised manuscript. If you need to cite a retracted article, indicate the article’s retracted status in the References list and also include a citation and full reference for the retraction notice.**

We have reviewed the reference list and confirmed that we do not cite any retracted articles. The adjustments made to the reference list are highlighted and referred to the required formatting. All cited references refer to corresponding citations throughout the manuscript text and they are complete and correct.

**Authors’ responses to the Editor (Email May 2^nd^, 2023)**

2. **Language and Abbreviations: The authors should improve the English writing and correct any errors that may be present. Additionally, abbreviations must be defined upon first appearance in the text. Non-standard abbreviations should be avoided unless they appear at least three times in the text, and their use should be kept to a minimum.**

We improved the English writing and corrected any errors that were present. We add the certificate of professional translation during the submission of the revised article. Abbreviations were kept to a minimum.

3. **Introduction: The introduction should be improved by justifying how gamification can be associated with the parameters that will be measured.**

The introduction was improved as requested.

4. **Methodology: The methodology should be reviewed to ensure that the risk of bias in the studies included is measured appropriately. The exclusion criteria should be clarified, and the research questions proposed should be clearly stated. The information should also be presented in a logical sequence.**

The methodology was reviewed to ensure that the risk of bias in the studies included is measured appropriately, as well we clarified the exclusion criteria and the research questions proposed.

5. **References: The references should be reviewed to ensure that they correspond to what is intended to be cited.**

We also reviewed the references to ensure that they correspond to what is intended to be cited.

**Authors’ responses to Journal requirements (Email May 2^nd^, 2023)**

6. **Please ensure that your manuscript meets PLOS ONE's style requirements, including those for file naming. The PLOS ONE style templates can be found at https://journals.plos.org/plosone/s/file?id=wjVg/PLOSOne_formatting_sample_main_body.pdf and https://journals.plos.org/plosone/s/file?id=ba62/PLOSOne_formatting_sample_title_authors_affiliations.pdf**

Our manuscript meets PLOS ONE's style requirements, and the PLOS ONE style templates were used.

7. **Please note that we require the PRISMA-P checklist as supplementary file to be provided with protocols for systematic reviews. Therefore, please remove the standard PRISMA checklist from your submission and provide a completed PRISMA-P checklist as supplementary file instead. For further information please refer to: https://www.prisma-statement.org/Extensions/Protocols. Thank you for your attention. We look forward to hearing from you.**

We provided the PRISMA-P checklist as supplementary file.

8. **We note that your article has been submitted as a "Registered Report Protocol" article type. Please note that your submission is not suitable for the article type "Registered Report Protocol". The "Registered Report Protocol" article type is only suitable for proposals of studies that have not yet started, and which will undergo editorial assessment per the Registered Reports framework. When resubmitting your manuscript, we ask that you update your article type to "Study Protocol" in the online submission form. Please note that some fields in the submission form, particularly in the "Additional Information" field, will have been reset with this change, so please go through your submission in full to ensure that all information is accurate and complete when resubmitting your manuscript.**

When resubmitting our manuscript, we updated our article type to "Study Protocol" in the online submission form.

9. **Thank you for stating the following financial disclosure:**

**“This study was partially financed by the Coordenação de Aperfeiçoamento de Pessoal de Nível Superior - Brasil (CAPES) - Finance Code 001”**

**Please state what role the funders took in the study. If the funders had no role, please state: "The funders had no role in study design, data collection and analysis, decision to publish, or preparation of the manuscript."**

**If this statement is not correct you must amend it as needed.**

We updated our Funding statement to: *This study was financed in part by the Coordenação de Aperfeiçoamento de Pessoal de Nível Superior - Brasil (CAPES) - Finance Code 001; and in part by the Ignacio H. Larramendi Research Grant granted by the Mapfre Foundation in 2022 to UCAM - Catholic University of Murcia for the "PECES" research project to Dr. Manuel Pardo Ríos and his research team. The funders had no role in study design, data collection and analysis, decision to publish, or preparation of the manuscript*.

10. **We note that you have stated that you will provide repository information for your data at acceptance. Should your manuscript be accepted for publication, we will hold it until you provide the relevant accession numbers or DOIs necessary to access your data. If you wish to make changes to your Data Availability statement, please describe these changes in your cover letter and we will update your Data Availability statement to reflect the information you provide.**

We updated our Data Availability statement to: All relevant data are within the paper and its Supporting Information files. No datasets were generated or analyzed during the current study.

**Authors’ Responses to Reviewers' comments (Email May 2^nd^, 2023)**

11. **Does the manuscript provide a valid rationale for the proposed study, with clearly identified and justified research questions?**

**The research question outlined is expected to address a valid academic problem or topic and contribute to the base of knowledge in the field.**

**Reviewer #1: No**

**Reviewer #2: Yes**

Considering the importance of making clear the justification for our study, as well as the research questions, we decided to add this information more clearly in the introduction of our manuscript. In addition, after the suggestions received, we made changes to highlight the innovative character of our study for an existing valid academic problem, as well as the contribution of the construction of this study to science. We hope we were able to clarify this information after reviewing the manuscript.
