# Supplementary material for: Gamification as a health education strategy of adolescents at school: Protocol for a systematic review and meta-analysis
Source: PLoS One. 2023 Nov 30;18(11):e0294894. doi: 10.1371/journal.pone.0294894 (PMC10688727; doi:10.1371/journal.pone.0294894)
Supplement: Attachment — Submitted filename: Response to Reviewers_R2.docx [file pone.0294894.s005.docx]

**Federal University of Rio Grande do Norte**

**Post-Graduation Program in Public Health**

Av. Senador Salgado Filho, 1787 – Lagoa Nova

ZIP: 59056-000 – Natal, RN, Brazil

September 14th, 2023

Rebuttal Letter for PLOS ONE *[PONE-D-23-04396R2] -[EMID:12c7612585d2c131]*

Dear M.Sc. Delfina Fernandes Hlashwayo,

Thank you for considering our manuscript for publication at PLOS ONE. We are certain of the credibility and commitment that PLOS ONE has to quality in science, and we hope that the modifications made in our manuscript fully meet the publication criteria.

We have read the reviewers' and editor’s comments carefully, answering all questions in this letter and made the requested changes in the revised manuscript. We are sure that the suggestions add quality and clarity to our paper. We hope the revised manuscript is now suitable for publication at PLOS ONE.

Once again, thank you for considering our manuscript and we are at your disposal for any further information.

Best regards,

The authors.

**Authors’ responses to the Editor**

1. **To enhance the ease of reference, we kindly request that you present the PRISMA-P checklist using page numbers instead of line numbers.**

*We appreciate your availability to revise our manuscript.* *We attended to your request and update PRISMA-P using page numbers.*

2. **While the strategy for Embase is present in the supplementary file, we recommend adding the search strategies for the following databases as well: MEDLINE, Scopus, ERIC, ScienceDirect, Web of Science, Cochrane, LILACS, APA, and ADOLEC.**

*We thank you for your considerations. PRISMA-P Statement recommends, we have to “specify all databases, registers, websites, organisations, reference lists, and other sources searched or consulted to identify studies. Specify the date when each source was last searched or consulted”.*

*We confirm that we will follow the Preferred Reporting Items for Systematic Reviews and Meta-Analyses Protocols (PRISMA-P) guidelines in developing our systematic review, but we have chosen to make it available in this protocol article, as a supplementary file, the precise and full search strategies for only one database (EMBASE) including any filters and limits used, as a way of guarding against a possible misuse of our search strategies while we are developing the systematic review.*

3. **The addition of a translation certificate as a supplementary file is not required. We kindly ask that you exclude it from the submission. Similarly, the funding disclosure should not be included as a supplementary file.**

*We thank you for this recommendation. We will exclude both translation certificate and funding* disclosure from the submission.

**Authors’ Responses to Reviewers' comments:**

1. **Does the manuscript provide a valid rationale for the proposed study, with clearly identified and justified research questions?**

**The research question outlined is expected to address a valid academic problem or topic and contribute to the base of knowledge in the field.**

**Reviewer #3: Yes**

**Reviewer #4: Partly**

*We appreciate your availability to revise our manuscript and all your comments. Considering the innovative nature of using gamification as a health education strategy for schoolchildren, we guided our research questions according to the dimensions to be explored in the construction of the review: food consumption, sleep and physical activity. The research questions, in addition to being in the registered PRISMA-P protocol, are fully described in the manuscript methodology, more specifically on page 5, lines 94-11, and they have been constructed in accordance with the justification presented in the Introduction section.*

2. **Is the protocol technically sound and planned in a manner that will lead to a meaningful outcome and allow testing the stated hypotheses?

   The manuscript should describe the methods in sufficient detail to prevent undisclosed flexibility in the experimental procedure or analysis pipeline, including sufficient outcome-neutral conditions (e.g. necessary controls, absence of floor or ceiling effects) to test the proposed hypotheses and a statistical power analysis where applicable. As there may be aspects of the methodology and analysis which can only be refined once the work is undertaken, authors should outline potential assumptions and explicitly describe what aspects of the proposed analyses, if any, are exploratory.**

**Reviewer #3: Yes**

**Reviewer #4: Partly**

*We thank you for your revision. As described in the manuscript, the protocol is based on the Preferred Reporting Items for Systematic Reviews and Meta-Analyses Protocols (PRISMA-P) guidelines, which can be found as supplementary material S1. We will follow all the necessary steps to develop the systematic review according to the described protocol, which was methodologically developed to allow us to test the hypotheses and answer the questions proposed in our study.*

*We hope that the results presented in the systematic review that will be developed based on this protocol can guide the creation of new health education tools for school adolescents using immersive technologies.*

3. **Is the methodology feasible and described in sufficient detail to allow the work to be replicable?**

**Descriptions of methods and materials in the protocol should be reported in sufficient detail for another researcher to reproduce all experiments and analyses. The protocol should describe the appropriate controls, sample size calculations, and replication needed to ensure that the data are robust and reproducible.**

**Reviewer #3: Yes**

**Reviewer #4: No**

*We appreciate your revision. We confirm that the protocol is based on the Preferred Reporting Items for Systematic Reviews and Meta-Analyses Protocols (PRISMA-P) guidelines and the final report will be developed following PRISMA and the Cochrane Handbook for Systematic Reviews of Interventions.*

*Therefore, we confirm that this protocol has enough details for another researcher to reproduce the review and analyses. Furthermore, the protocol contains a description of all eligibility criteria, the selection steps that will be followed, the data that will be extracted and the possible analyzes to be carried out. Therefore, it is a protocol that can be reproduced.*

4. **Have the authors described where all data underlying the findings will be made available when the study is complete?**

**The [PLOS Data policy](https://journals.plos.org/plosone/s/materials-and-software-sharing) requires authors to make all data underlying the findings described in their manuscript fully available without restriction, with rare exception, at the time of publication. The data should be provided as part of the manuscript or its supporting information, or deposited to a public repository. For example, in addition to summary statistics, the data points behind means, medians and variance measures should be available. If there are restrictions on publicly sharing data—e.g. participant privacy or use of data from a third party—those must be specified.**

**Reviewer #3: Yes**

**Reviewer #4: Yes**

*We thank you for your revision. All information related to the construction of the article to be published was made available in the files added in the submission.*

5. **Is the manuscript presented in an intelligible fashion and written in standard English?**

**PLOS ONE does not copyedit accepted manuscripts, so the language in submitted articles must be clear, correct, and unambiguous. Any typographical or grammatical errors should be corrected at revision, so please note any specific errors here.**

**Reviewer #3: Yes**

**Reviewer #4: No**

*Thank you for your comments. We reviewed the translation of our article with a professional translator.*

6. **Review Comments to the Author**

**Please use the space provided to explain your answers to the questions above and, if applicable, provide comments about issues authors must address before this protocol can be accepted for publication. You may also include additional comments for the author, including concerns about research or publication ethics.

You may also provide optional suggestions and comments to authors that they might find helpful in planning their study.


(Please upload your review as an attachment if it exceeds 20,000 characters)**

**Reviewer #3: The authors carefully responded to all requests from reviewers.

In general, the study's proposal is innovative and will bring contributions to teaching and learning, as well as future research and university extension actions for adolescents.**

**The introduction is well delimited and now well justified in relation to the purpose of the protocol. The authors propose a search in several databases, which may help to overcome a study limitation of finding research to compose the study, as long as the theme is relatively new and directed only to adolescents.**

**Some notes:**

**In eligibility criteria: It must be clear in the protocol whether students up to high school or college or technical students will be included (In some countries there are technical education courses). As a result of in the first semesters of college or technical courses most are composed by teenagers.
The suggestion is that the approach be taken regardless of the level of schooling in progress, as data to be extracted and analyzed.**

**Reviewer #4: It is interesting work but not entirely clear to the reader. The abstract was well written but I could not understand it fully despite reading the main manuscript 2-3 times.**

*We thank you for all the comments and evaluations presented. We always do our best to respond to suggestions and increase the quality of our work.*

*The amount of database is extensive so that our search is more comprehensive, and we have a better chance of finding articles that can answer our research questions.*

*We thank you for the suggestion regarding the study locations of the adolescents included in the studies and made an adjustment to the eligibility criteria (highlighted on page 6 of the manuscript)*

*To make our introduction more understandable, we made some adjustments.*

7. **PLOS authors have the option to publish the peer review history of their article (**[**what does this mean?**](https://journals.plos.org/plosone/s/editorial-and-peer-review-process#loc-peer-review-history)**). If published, this will include your full peer review and any attached files.**

**If you choose “no”, your identity will remain anonymous but your review may still be made public.**

**Do you want your identity to be public for this peer review? For information about this choice, including consent withdrawal, please see our** [**Privacy Policy**](https://www.plos.org/privacy-policy)**.**

**Reviewer #3: Yes: Flaviana Vely Mendonça Vieira**

**Reviewer #4: No**

*We are grateful for all the contributions presented by both reviewers to improve the construction of our article.*
