# Supplementary material for: Gamification as a health education strategy of adolescents at school: Protocol for a systematic review and meta-analysis
Source: PLoS One. 2023 Nov 30;18(11):e0294894. doi: 10.1371/journal.pone.0294894 (PMC10688727; doi:10.1371/journal.pone.0294894)
Supplement: Attachment — Submitted filename: Response to Reviewers PONE-D-23-04396R3.docx [file pone.0294894.s006.docx]

**Federal University of Rio Grande do Norte**

**Post-Graduation Program in Public Health**

Av. Senador Salgado Filho, 1787 – Lagoa Nova

ZIP: 59056-000 – Natal, RN, Brazil

November 7^th^, 2023

Rebuttal Letter for PLOS ONE *[PONE-D-23-04396R3]*

Dear M.Sc. Delfina Fernandes Hlashwayo,

Thank you for considering our manuscript for publication at PLOS ONE. We are certain of the credibility and commitment that PLOS ONE has to quality in science, and we hope that the modifications made in our manuscript fully meet the publication criteria.

We have read the reviewer's and editor’s comments carefully, answering all questions in this letter and made the requested changes in the revised manuscript. We are sure that the suggestions add quality and clarity to our paper. We hope the revised manuscript is now suitable for publication at PLOS ONE.

Once again, thank you for considering our manuscript and we are at your disposal for any further information.

Best regards,

The authors.

**Authors’ responses to the Editor**

1. **Ensure to check for any spelling, grammar, or phrasing errors.**

*We appreciate your availability to revise our manuscript.* *We attended to your request, and we revised all the text.*

2. **When referring to the level of education (high school, college, or technical school), please avoid using the term "place of study."**

*We thank you for your considerations and we have made the changes.*

3. **Review the planned start and end dates, as the protocol has not yet been published. The registered start and end dates are January 10th, 2023, and September 8th, 2023, which have passed.**

*We thank you for this recommendation. We reviewed the planned start and end dates in the protocol.*

4. **Kindly revise the text in the methodology section where it is written in the past tense (line 129).**

*We appreciate your considerations and we have reviewed the text.*

5. **Please ensure the correct naming of Supplementary Files.**

*We thank you for this recommendation. We reviewed the names of Supplementary Files.*

6. **In line 201, please provide the complete name of the program. Additionally, in line 202, consider using the correct symbol for the chi-square test or write it out in full.**

*We appreciate your considerations. We provided the information required.*

7. **Please avoid defining the included population as "population used" (line 287).**

*We are grateful for your recommendations. All suggestions were very important to improve the quality of our study.*

**Authors’ Responses to Reviewers' comments:**

1. **Does the manuscript provide a valid rationale for the proposed study, with clearly identified and justified research questions? The research question outlined is expected to address a valid academic problem or topic and contribute to the base of knowledge in the field.**

**Reviewer #3: Yes**

*We thank you for all the comments and evaluations presented. We always do our best to respond to suggestions and increase the quality of our work.*

2. **Is the protocol technically sound and planned in a manner that will lead to a meaningful outcome and allow testing the stated hypotheses? The manuscript should describe the methods in sufficient detail to prevent undisclosed flexibility in the experimental procedure or analysis pipeline, including sufficient outcome-neutral conditions (e.g. necessary controls, absence of floor or ceiling effects) to test the proposed hypotheses and a statistical power analysis where applicable. As there may be aspects of the methodology and analysis which can only be refined once the work is undertaken, authors should outline potential assumptions and explicitly describe what aspects of the proposed analyses, if any, are exploratory.**

**Reviewer #3: Yes**

*We appreciate your revision.*

3. **Is the methodology feasible and described in sufficient detail to allow the work to be replicable? Descriptions of methods and materials in the protocol should be reported in sufficient detail for another researcher to reproduce all experiments and analyses. The protocol should describe the appropriate controls, sample size calculations, and replication needed to ensure that the data are robust and reproducible.**

**Reviewer #3: Yes**

*We appreciate your revision, and we confirm that this protocol can be reproduced.*

4. **Have the authors described where all data underlying the findings will be made available when the study is complete? The [PLOS Data policy](https://journals.plos.org/plosone/s/materials-and-software-sharing) requires authors to make all data underlying the findings described in their manuscript fully available without restriction, with rare exception, at the time of publication. The data should be provided as part of the manuscript or its supporting information, or deposited to a public repository. For example, in addition to summary statistics, the data points behind means, medians and variance measures should be available. If there are restrictions on publicly sharing data—e.g. participant privacy or use of data from a third party—those must be specified.**

**Reviewer #3: Yes**

*We are grateful for your revision. All information related to the construction of the article to be published was made available in the files added in the submission.*

5. **Is the manuscript presented in an intelligible fashion and written in standard English? PLOS ONE does not copyedit accepted manuscripts, so the language in submitted articles must be clear, correct, and unambiguous. Any typographical or grammatical errors should be corrected at revision, so please note any specific errors here.**

**Reviewer #3: Yes**

*Thank you for your comments. We reviewed the translation of our article with a professional translator.*

6. **Review Comments to the Author. Please use the space provided to explain your answers to the questions above and, if applicable, provide comments about issues authors must address before this protocol can be accepted for publication. You may also include additional comments for the author, including concerns about research or publication ethics.
   You may also provide optional suggestions and comments to authors that they might find helpful in planning their study. (Please upload your review as an attachment if it exceeds 20,000 characters)**

**Reviewer #3: I appreciate the opportunity to evaluate this study, which makes an important contribution to the area.
The authors made the indicated adjustments.**

*We appreciate your availability to revise our manuscript and all your comments. We hope that the results presented in the systematic review that will be developed based on this protocol can be an instrument on health education for school adolescents.*

7. **PLOS authors have the option to publish the peer review history of their article ([what does this mean?](https://journals.plos.org/plosone/s/editorial-and-peer-review-process#loc-peer-review-history)). If published, this will include your full peer review and any attached files. If you choose “no”, your identity will remain anonymous but your review may still be made public. Do you want your identity to be public for this peer review? For information about this choice, including consent withdrawal, please see our** [**Privacy Policy**](https://www.plos.org/privacy-policy)**.**

**Reviewer #3: No**

*We are grateful for all your contributions presented to improve the construction of our article.*
